# Near-Infrared Spectroscopy during the Verbal Fluency Task before and after Treatment with Image Exposure and SSRI Therapy in Patients with Obsessive-Compulsive Disorder

**DOI:** 10.1155/2014/591023

**Published:** 2014-09-16

**Authors:** Mari Nakanishi, Harumi Oshita, Yoshihiro Tanaka, Ayako Inoue, Chiwa Kawashima, Kana Okamoto, Shunsuke Kobayashi, Yoshinobu Ishitobi, Taiga Ninomiya, Jotaro Akiyoshi

**Affiliations:** ^1^Department of Neuropsychiatry, Oita University Faculty of Medicine, Hasama-Machi, Oita 879-5593, Japan; ^2^Department of Applied Linguistics, Oita University Faculty of Medicine, Hasama-Machi, Oita 879-5593, Japan

## Abstract

Drug therapy with selective serotonin reuptake inhibitors (SSRIs) has been used as a treatment for obsessive-compulsive disorder (OCD). In the present case report, exposure therapy was used in addition to escitalopram (20 mg) to treat a 28-year-old female patient with OCD for 6 months. Her obsessive-compulsive symptoms comprised thoughts of words such as rape, crematorium, neck hanging, unhappy, death, die, and kill and images such as a shelf of gods, a shrine, a Buddhist altar, the sun, the sky, and the faces of her parents, siblings, and relatives. As exposure therapy, she was asked to view the images associated with these symptoms three times a day along with drug therapy. With the combination of drug and exposure therapies, her obsessive-compulsive symptoms improved within 6 months, with no interference in her daily life. Multichannel near-infrared spectroscopy (NIRS) showed improvement of brain function in the temporal and frontal lobes after treatment. These results suggest that NIRS can be used as an indicator of brain function improvement in patients with OCD.

## 1. Introduction

Obsessive-compulsive disorder (OCD), which is one of the most common mental disorders, has a reported frequency of 1.9%–3.3% [1, 2]. Patients with OCD exhibit recurrent and persistent thoughts and urges or images that they try to suppress or ignore. As a result, the social and occupational functioning of OCD patients is impaired [3]. Cognitive-behavioral therapy (CBT) and selective serotonin reuptake inhibitors (SSRIs) have long been recognized as proven treatments for OCD [4, 5]. However, CBT is less commonly used as compared with SSRI treatment [6]. Moreover, about 60% of the patients who have been treated with CBT did not receive the minimum standard of CBT [7]. Research on the clinical features of OCD is progressing, but studies on the mechanisms underlying the biological etiology of OCD are not sufficiently advanced. Brain imaging techniques, such as positron emission tomography (PET), single photon emission computed tomography (SPECT), and functional magnetic resonance imaging (fMRI), have been used to study the pathogenesis of OCD [8–10]. Recently, multichannel near-infrared spectroscopy (NIRS) has been introduced as a new brain imaging technique. This system has a high resolution, and measurements can be made easily and noninvasively at the bedside [11]. It operates by measuring the changes in the concentrations of oxygenated and deoxygenated hemoglobin in the brain.

## 2. Case Report

**Figure 1:**
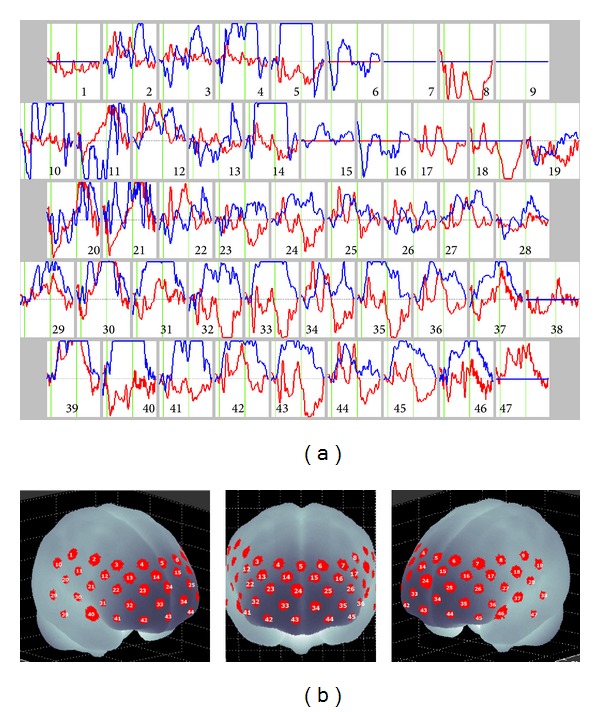
(a) Grand averaged waveforms of [oxy-Hb] during verbal fluency task (between two green vertical lines in each graph) in 47 channels over the frontal and temporal regions measured by NIRS. Red and blue lines represent pretreatment and posttreatment, respectively. (b) The 47 measuring areas are labeled CH1 to CH47 from the right posterior to the left anterior.

A 28-year-old female from another prefecture came to our department for psychiatric diagnosis and treatment. Because her obstetrician informed her that her husband's sperm and her egg were old given their age, she worried about the health of her fetus during pregnancy. After reading a newspaper article on Down syndrome, she began to have thoughts about the term Down syndrome. When she thought of it, she subsequently and repeatedly thought of the word healthy until she canceled the term Down syndrome. She was diagnosed with OCD in a psychiatric hospital at the age of 26, but she refused to be treated because she believed that she could cure the OCD with her own efforts. When she was 27 years old, the words unhappy, death, die, and kill came to her mind at the same time as the face of an acquaintance. She tried to counteract these obsessive-compulsive thoughts by repeatedly thinking of the words different and not die. After four months, she began to think of the words rape, crematorium, neck hanging, unhappy, death, die, and kill and then images of a shelf of gods, a shrine, a Buddhist altar, the sun, and the sky. She tried to avoid looking at these things. She visualized the faces of her parents, siblings, and relatives appearing in her images at the same time as the images of these words. She tried to erase the words and images by repeating the word different. Because her obsessive-compulsive symptoms did not improve, she revisited the psychiatric hospital and was diagnosed with OCD again. Treatment with aripiprazole and fluvoxamine was initiated. She had received psychoanalytic counseling from a psychiatrist for 6 months, but she did not experience relief. She discontinued the psychoanalytic counseling and drug treatment after consultation with her doctor. Next, she visited our department with the hope of improving her obsessive-compulsive symptoms. She was diagnosed with OCD according to the Diagnostic and Statistical Manual for Mental Disorders (fifth edition) on the basis of her recurrent thoughts and images and her attempts to suppress them. We excluded current Axis I diagnoses such as major depressive disorder using the Mini International Neuropsychiatry Interview (MINI). She was prescribed escitalopram (20 mg for 6 months) and CBT. She was asked to look at the images of a shelf of gods, a shrine, a Buddhist altar, the sun, the sky, and the faces of her parents, siblings, and relatives three times a day. After 6 months of these treatments, her obsessive-compulsive symptoms improved to the extent that they did not bother the patient. After 6 months, she could begin the qualification tests and do housework such as cooking, washing, and cleaning. Our treatment was successful, and the patient showed clinically significant changes in her obsessive-compulsive symptoms. The first treatment was probably not adequate, and after initiating an adequate treatment the patient fully responded. The Yale-Brown Obsessive Compulsive Scale score reduced from 31 points to 10 points within 6 months of treatment. Changes in [oxy-Hb] were measured as an index of changes in cerebral blood volume and in [deoxy-Hb] using a 47-channel NIRS machine (Hitachi ETG-4000; Hitachi Medical Systems, Tokyo, Japan). The correspondence between NIRS channels and measurement points on the cerebral cortex was confirmed by comparison with the results of a multiparticipant study of anatomical craniocerebral correlation [12]. We used verbal fluency task in the phonemic variant. NIRS results showed increased oxygenated hemoglobin levels in the frontal and temporal lobes after 6 months (Figure 1).

## 3. Discussion

The present result is consistent with the results of previous NIRS studies that patients with OCD showed decreased prefrontal hemodynamic responses [13, 14]. In this patient with OCD, SSRI treatment with exposure therapy was symptomatically effective, and NIRS findings showed improvement of brain function in the temporal and frontal lobes after treatment. This suggests that SSRIs and exposure therapy improve the function of the frontal and temporal lobes in OCD. SSRI has already established its efficacy in obsessive-compulsive disorder [15]. OCD has been attributed to dysfunction in the interaction between basal ganglia and the cerebral cortex, specifically the lack of cortical control over the striatum [10]. Thus, the basal ganglia lacking inhibitory control from the upper center become hyperactive, resulting in the psychiatric symptoms of OCD. Antidepressants are one of the drugs that have been efficacious in OCD. Amongst them, SSRIs have recently been used as the first line of treatment. This is because of the lack of side effects and the beneficial effects; however, SSRIs are not effective in 30%–50% of patients with OCD [16]. Therefore, additional therapy with CBT is necessary for treating OCD [17]. Exposure therapy with images, as in this case, has been recommended for the treatment of patients with OCD. Although the behavioral constraints required from subjects are smaller in NIRS as compared with PET, fMRI, and SPECT, very few NIRS studies have been performed on patients with OCD. The disadvantage of NIRS is that it cannot measure the function of the deep parts of the brain. Because NIRS is convenient, it has been recommended for use in a wide range of brain research studies. NIRS showed improvement of brain function in the temporal and frontal lobes after SSRI therapy with image exposure therapy. As the waveforms of monopolar depression and schizophrenia showed low oxygenated hemoglobin level, the clinical difference between OCD and other psychiatric disorders is difficult [18].

## 4. Conclusion

SSRI therapy combined with exposure therapy in patients with OCD is effective and is associated with functional improvement in the frontal and temporal lobes.
